# Microbial metabolite butyrate promotes induction of IL-10^+^IgM^+^ plasma cells

**DOI:** 10.1371/journal.pone.0266071

**Published:** 2022-03-25

**Authors:** Bandik Föh, Jana Sophia Buhre, Hanna B. Lunding, Maria E. Moreno-Fernandez, Peter König, Christian Sina, Senad Divanovic, Marc Ehlers

**Affiliations:** 1 Institute of Nutritional Medicine, University of Lübeck and University Hospital Schleswig-Holstein, Lübeck, Germany; 2 Department of Medicine I, University Hospital Schleswig-Holstein, Lübeck, Germany; 3 Division of Immunobiology, Cincinnati Children’s Hospital Medical Center, Cincinnati, OH, United States of America; 4 Institute of Anatomy, University of Lübeck, Lübeck, Germany; 5 Department of Pediatrics, University of Cincinnati College of Medicine, Cincinnati, OH, United States of America; 6 Center for Inflammation and Tolerance, Cincinnati Children’s Hospital Medical Center, Cincinnati, Ohio, United States of America; 7 Airway Research Center North, University of Lübeck, German Center for Lung Research (DZL), Lübeck, Germany; Lewis Katz School of Medicine, Temple University, UNITED STATES

## Abstract

The microbially-derived short-chain fatty acid butyrate is a central inhibitor of inflammatory innate and adaptive immune responses. Emerging evidence suggests that butyrate induces differentiation of IL-10-producing (IL-10^+^) regulatory B cells. However, the underlying mechanisms of butyrate-driven modulation of B cell differentiation are not fully defined. Given the dominant role of regulatory plasma cells (PCs) as the main source of anti-inflammatory cytokines including IL-10 and the observation that butyrate also induces the differentiation of PCs, we here investigated the effect of the microbial metabolite butyrate on the induction of regulatory IL-10^+^ PCs and underlying mechanisms. Here we show that butyrate induces the differentiation of IL-10^+^IgM^+^ PCs. *Ex vivo*, butyrate, but hardly propionate, another microbially-derived short-chain fatty acid, induced the differentiation of IL-10^+^IgM^+^ CD138^high^ PCs from isolated splenic murine B cells. *In vivo*, administration of butyrate via drinking water or by daily intraperitoneal injection increased the number of IL-10^+^IgM^+^ CD138^high^ PCs in the spleens of Ovalbumin (Ova)/complete Freund’s adjuvant-immunized mice. The induction of these regulatory PCs was associated with an increase of anti-Ova IgM, but a reduction of anti-Ova class-switched pathogenic IgG2b serum antibodies. Based on the knowledge that butyrate inhibits histone deacetylases (HDACs) thereby increasing histone acetylation, we identified here that HDAC3 inhibition was sufficient to induce PC differentiation and IL-10^+^ expression. Furthermore, reduced mitochondrial superoxide levels following butyrate treatment and HDAC3 inhibition were necessary for PC differentiation, but not IL-10 expression. In summary, the microbial metabolite butyrate promotes the differentiation of IgM^+^ PCs and their expression of IL-10. HDAC3 inhibition may be involved as an underlying pathway for both PC differentiation and IL-10 expression, while reduced mitochondrial superoxide levels are crucial only for PC differentiation. The induction of regulatory IL-10^+^IgM^+^ PCs and the inhibition of class switching to antigen-specific pathogenic IgG subclasses might represent important pathways of butyrate to limit inflammation.

## Introduction

Gut microbiome and the host’s immune system are tightly intertwined in a complex network of reciprocal regulatory mechanisms in health and disease [1, 2]. Microbially-derived metabolites are of crucial relevance in regulating immune homeostasis and protecting against allergy and autoimmunity [3–5]. Accordingly, gut microbial composition and microbial metabolites are frequently disturbed in autoimmune and allergic diseases [1, 4, 6, 7].

Short-chain fatty acids (SCFAs), including butyrate (BA; C4) and propionate (PA; C3), are potent microbial metabolites with a wide array of immunomodulatory functions [3, 5]. SCFAs are produced mainly from dietary fiber (undigested complex carbohydrates) by microbial fermentation and reach high concentrations of 60–140 mM in the colon, whereas lower concentrations are detected in the blood (micromolar range) [8, 9]. Importantly, SCFAs accumulate in tissues of high immunological relevance, such as mesenteric lymph nodes and the spleen, reaching concentrations between 1 and 1.5 mmol*kg^-1^ in the case of BA [9]. Furthermore, oral administration of SCFAs is an effective method for increasing SCFA concentrations in these organs [9]. Thus, immunological effects of SCFAs are more likely to occur either in the gastrointestinal tract or in immunological effector organs where high concentrations of SCFAs are present rather than in the systemic circulation.

Besides acting as highly abundant energy substrates promoting intestinal barrier function [10, 11], SCFAs also act as signaling molecules via the activation of G-protein-coupled receptor 41 (GPR41), GPR43 and GPR109a [3]. By activating GPRs, SCFAs induce neutrophil chemotaxis [12, 13] and differentiation of peripheral regulatory T cells (Tregs) [14, 15] protecting against experimental colitis [14, 15], food allergy [16], and allergic asthma [17]. In addition, SCFAs regulate immune functions by inhibiting histone deacetylases (HDACs). HDAC inhibition by SCFAs takes part in the induction of peripheral Tregs [18, 19], and follicular regulatory T cells [20], and is responsible for the inhibition of pro-inflammatory cytokine production in peripheral mononuclear blood cells [21], neutrophils [22], macrophages [23], dendritic cells [17, 24], effector CD4^+^ T cells [25], and B cells [9, 26, 27].

Considering the anti-inflammatory effects of SCFAs on a wide array of immune cells, modulation of the B cell compartment by SCFAs has not been fully defined [1, 28]. B cells, in part due to the production of pathogenic IgG (auto-) or IgE antibodies and activation of other immune cells, are central to the pathogenesis of autoimmunity and allergy, respectively [29]. Moreover, B cell subsets, including CD138^high^ plasma cells (PCs), are increasingly recognized to exert anti-inflammatory functions by producing anti-inflammatory cytokines (IL-10, TGFβ, IL-35) [30–32] and highly glycosylated (esp. sialylated) IgG antibodies [33–38]. Thus, modulating the effector B cell phenotype towards a more anti-inflammatory state represents an intriguing target for the treatment of autoimmune and allergic diseases. Notably, BA protects against autoimmunity by increasing IL-10^+^ Bregs [25, 27] and by supporting antibody responses both in the intestine and systemically [9, 39]. However, whether induction of regulatory properties of B cells and induction of PC differentiation by BA are functionally and mechanistically related and which cellular pathways are substantially involved remains to be defined.

Here we investigated the effects of BA on PC differentiation and the expression of anti-inflammatory cytokines, in particular IL-10. We show that BA increases the differentiation of IL-10^+^IgM^+^ PCs both *ex vivo* and *in vivo* which was associated with a reduction of antigen-specific IgG2b class switching. Mechanistically, BA led to increased histone acetylation in B cells, which is a downstream effect of HDAC inhibition, and inhibition of HDAC3 was identified to be sufficient for the induction of PCs and IL-10 expression underlining the importance of epigenetic modulation of B cell function. Reduced mitochondrial superoxide levels after BA treatment and following HDAC3 inhibition were necessary for PC differentiation, but not for IL-10 expression, indicating that these processes are initiated in parallel and not in consequence of one another.

## Materials and methods

### Reagents

Ovalbumin (Ova) was purchased from Sigma-Aldrich (USA). Complete Freund’s adjuvant (CFA) was prepared by adding heat-killed Mtb.H37 RA (BD Biosciences, CA, USA) to incomplete Freund’s adjuvant (IFA; Sigma-Aldrich, Germany; 1 mg Mtb/ml). 2-Deoxy-D-glucose (2-DG, #D8375), DMSO (#S-002-M), phorbol-12-myristate-13-acetate (PMA, #P1585), HDAC3-inhibitor RGFP966 (#SML1652), sodium butyrate (BA, #303410), sodium propionate (PA, #P1880), trichostatin A (TSA, #T8552), and Lipopolysaccharides (LPS) were all purchased from Sigma-Aldrich (USA). Selective GPR43/FFAR2 agonist (S-2-(4-chlorophenyl)-3,3-dimethyl-N-(5-phenylthiazol-2-yl)butanamide, #371725) was purchased from Merck (Germany). Sodium butyrate (BA) for application in animals was freshly prepared for every use by dilution with sterile PBS for i.p. injections or designated drinking water from the animal facility. Sodium butyrate solution (BA, #TR-1008-G) for cell culture experiments was purchased from Merck (Germany) and working solutions were freshly prepared. FACS-buffer (2% fetal calf serum (FCS) or bovine serum albumin (BSA) and 0.01% NaN in PBS) was freshly prepared at the laboratory.

### Mice

C57BL/6 wt mice (WT) were purchased from Charles River Laboratories (Bar Harbor, ME, USA). IL-10 GFP reporter (Vert-X) mice were originally provided by Prof. Christopher L. Karp (Cincinnati Children’s Hospital Medical Center, Cincinnati, OH, USA) and bred in-house [40]. Mice were kept at the local animal facilities of either the University of Luebeck or Cincinnati Children’s Hospital Medical Center (Cincinnati, OH, USA) under specific-pathogen-free conditions at 22°C with a 12h light/dark cycle and with food and water *ad libitum*. Mice were kept and all experiments were performed in strict accordance with governmental and institutional guidelines with approval by the governmental administration and the animal research ethics boards of the corresponding ministries of the state of Schleswig-Holstein, Germany (approval number: 51-5/2019) or the Cincinnati Children’s Hospital Medical Center’s Institutional Animal Care and Use Committee (IACUC 2020–0034). Monitoring of the health and well-being of the mice was conducted at least daily by a qualified person. 8- to 12-week-old mice were used for *in vivo* experiments. 8- to 16-week-old mice were used for *ex vivo* experiments. All efforts were made to minimize and alleviate distress and suffering. Refined aspects of study planning, housing, and husbandry were applied. Power analysis was conducted prior to the study to reduce the number of animals to the necessary minimum for reliable results. No harmful noise, vibrations, or lighting were present during the study. Habituation and husbandry handling was combined with handling for research purposes wherever possible. Mice were anesthetized and analgized by injection of ketamine hydrochloride (80 mg/kg body weight; Medistar, Germany) and xylazine hydrochloride (10 mg/kg body weight; Bayer, Germany). Mice were sacrificed according to the respective governmental and institutional guidelines either by cervical dislocation or CO_2_ exposure.

### B cell isolation using magnetic activated cell sorting

B cells were isolated from murine spleens by depleting non-B cells using magnetic activated cell sorting (Mouse B cell isolation kit, #130-090-862, Miltenyi Biotec, Germany) following the manufacturer’s protocol. In short, splenic cells were obtained by passing the organ twice through 70 μm nylon filters (352350, Fisher Scientific, Germany). Non-B cells were labeled with biotinylated antibodies directed against several surface antigens that are not expressed by murine B cells (CD43, CD4, and Ter-119). Magnetic beads conjugated with anti-biotin antibodies were added as a secondary labeling agent. The cell suspension was then added to separation columns (LS columns, #130-042-401, Miltenyi Biotec, Germany) in a strong magnetic field (QuadroMACS Separator, #130-090-862, Miltenyi Biotec, Germany) capturing cells labeled with biotinylated antibodies and bound to magnetic anti-biotin beads. The non-labeled B cells were collected with the flow-through achieving a B cell purity of min. 95%.

### Cell culture

Isolated B cells were cultured in RPMI medium with 10% FCS, 100 μM 2-mercaptoethanol, 1 mM HEPES, and 100 U/ml Penicillin/Streptomycin (later referred to as *B cell medium*). B cells were cultured at 10^5^ to 10^6^ cells per ml. For basic PC-inducing conditions in all cell culture experiments, the medium was supplemented with 3 μg/ml LPS plus 10 ng/ml IL-6 (#216–16, Peprotech, USA) plus 100 nM all-trans Retinoic Acid (#R2625, Sigma-Aldrich, USA). Sodium butyrate (BA) and sodium propionate (PA) working solutions were freshly prepared and added directly to cell culture on day 0 of the experiments to final concentrations of 0.1 mM to 0.5 mM. 2-DG (200 μM), PMA (100 ng/ml), RGFP966 (10 μM), TSA (90 nM), and GPR43/FFAR2 agonist (1 μM) working solutions were freshly prepared and added directly to cell culture on day 0 of the experiments to the indicated final concentrations. Cells were cultured at 37°C and 5% CO_2_ (Heracell VIOS 160i, Thermo Fisher Scientific, USA) for up to four days as described for each experiment.

### Immunization and BA treatment in vivo

8 to 12-week-old WT or IL-10-reporter mice were treated with BA and immunized i.p. with 100 μg Ova/CFA. In brief, BA or the respective control was administered to the mice continuously starting 7 days before immunization in two different routes of administration: i) BA was dissolved in designated drinking water (DW) from the animal facility at 150 mM and provided *ad libitum*, DW was used as the control; ii) BA was administered by daily intraperitoneal (i.p.) injection (100 mg/kg body weight); PBS was injected as the vehicle control. Mice were weighed every 2–3 days and in the case of DW experiments, the consumption of water was recorded. 12 days after Ova/CFA immunization all mice were sacrificed, and spleens and sera were sampled for further analysis.

### Flow cytometry

Single-cell suspensions from cell culture were prepared by washing the cells twice (5 min, 400 x g, 4°C) and resuspending them in FACS buffer. Single-cell suspensions from murine spleens were prepared by passing the organ through a 70 μm nylon filter (#352350, Thermo Fisher Scientific, Germany), followed by incubation in ammonium-chloride-potassium lysing buffer (#A1049201, Thermo Fisher Scientific, USA) for 5 min at room temperature to remove erythrocytes. Afterward, cells were washed in FACS-buffer and passed again through a 70 μm nylon filter to remove any remaining tissue debris or cellular aggregates. The numbers of viable cells were determined and normalized to 0.2 to 1 x 10^6^ cells per sample for cell culture experiments before cell staining commenced.

For mitochondrial staining 0.2 x 10^6^ cells were seeded into V-shaped 96-well microplates and washed with 200 μl of FACS-buffer (5 min, 400 x g, 4°C). 100 μl staining solution containing RPMI and mitochondrial stains (see below) were added to the cells. After mixing the well by gently pipetting up and down, plates were incubated at 37°C in the dark for 15 minutes and washed with 300 μl of FACS-buffer. Three different fluorescent dyes (all Thermo Fisher Scientific, USA) were used: MitoTracker Green FM (#M7514, 100 nM), TMRE (#T669, 150 nM), and/or MitoSOX Red (#M36008, 5 μM). For extracellular staining, cells were seeded into V-shaped 96-well microplates and washed with 200 μl of FACS-buffer (5 min, 400 x g, 4°C). Subsequently, 100 μl staining solution containing FACS-buffer and fluorophore-conjugated antibodies were added to the cells in previously optimized concentrations. For additional intracellular staining, the samples were subsequently fixed with 200 μl Cytofix/Cytoperm for 30 min at room temperature according to the manufacturer’s instructions (#554722, BD Biosciences, USA) followed by permeabilization with Permeabilization Wash Buffer (#421002, Biolegend, USA), followed by resuspension in 100 μl staining solution containing Permeabilization Wash Buffer and fluorophore-conjugated-antibodies (45 min). The following fluorophore-coupled antibodies and reagents were used for extra- and/or intracellular stainings: BV786 Rat Anti-Mouse CD45R/B220 (monoclonal, #563894, clone RA3-6B2 (RUO), Thermo Fisher Scientific, USA), BV711 Rat Anti-Mouse CD138 (monoclonal, #563193, clone 281–2 (RUO), Thermo Fisher Scientific, USA), and PE-Cyanine7 Rat Anti-Mouse IgM (monoclonal, #25-5890-82, clone eB121-15F9, Thermo Fisher Scientific, USA). AF647-coupled Ova was used (#034784, Thermo Fisher Scientific, USA) to determine antigen-specific B cells. Anti-Mouse H3K27ac-antibody (monoclonal, #8173, Cell Signaling Technology, USA) was fluorophore-conjugated with Alexa Fluor 488 for flow cytometry using a commercially available kit following the manufacturer’s instruction (#A20181, Thermo Fisher Scientific, USA). eBioscience Fixable Viability Dye eFluor 780 was used for cell viability staining (#65-0865-14, Thermo Fisher Scientific, MA, USA). Cells were washed three times in 200 μl FACS-buffer (5 min, 400 x g, 4°C) after staining to remove excess dyes and/or antibodies. All fluorophore-conjugated antibodies, fluorescent dyes, and cells expressing fluorescent-reporter proteins were protected from light during the process of harvesting, staining, and measurement to ensure the least possible loss of fluorescent signal. Cells were always measured immediately after staining. Flow cytometric data were collected using either AttuneNXT (Thermo Fisher Scientific, USA) or LSRFortessa (BD Biosciences, USA), and data analysis was performed using FlowJo X software (vX0.7).

### qRT-PCR

Cultured B cells were lysed and RNA was extracted using the innuPREP RNA Mini Kit 2.0 (Analytik Jena, Germany) according to the manufacturer’s instructions and treated with amplification grade DNase I (Thermo Fisher Scientific, USA). RNA contents were quantified using a Nanodrop 2000 (Thermo Fisher Scientific, MA, USA) and stored at -80°C until further usage. 1 μg of isolated RNA per sample was added to RNase/DNase-free reaction tubes, and RNase/DNase-free water was added up to a volume of 11.5 μl. Next, 8.5 μl of freshly prepared Master Mix were added to each sample, containing 0.5 μl RNase inhibitor (Ribolock, 40 U/μl, #EO0384, Thermo Fisher, USA), 2 μl dNTP mix (dNTP set, #20–2011, 100 mM each, peqGOLD, VWR International, USA), 1 μl reverse transcriptase (RevertAid H Minus Reverse Transcriptase, #EP0442, 200 U/μL, Thermo Fisher, USA), 4 μl 5x Reaction Buffer (included in RevertAid H Minus Reverse Transcriptase Kit, #EP0442, Thermo Fisher, USA), and 1 μl Oligo(dT) (biomers.net, Germany). The reaction mix was incubated for 60 min at 42°C for reverse transcription, 10 min at 70°C for enzyme inactivation, diluted 1:5 in DNase-free water, and stored at -20°C until further usage. 2 μl cDNA sample per technical replicate were added to a 96-well PCR plate (Sarstedt, Germany). 10 μl PerfeCTa SYBR Green SuperMix (#733–1251, VWR International, USA) as well as 0.25 μl forward and reverse primers (final concentration of 125 nM each) were added following manufacturer’s instructions. 7.5 μl DNAse-free water was added for a final reaction volume of 20 μl per well. The primer pairs were designed using NCBI Primer-BLAST (https://www.ncbi.nlm.nih.gov/tools/primer-blast/), purchased from Metabion (Germany) and are shown in **Table 1**. The StepOnePlus Real-Time PCR System and Software (Thermo Fisher Scientific, USA) were used to conduct the qPCR following the manufacturer’s recommendations. At least two technical replicates were included for each sample. mRNA expression of each gene was compared to β-actin expression (endogenous housekeeping gene control) using the ddCT method and calculated as fold change of control.

**Table 1:** qRT-PCR primers. Gene targets, forward and reverse RT-PCR primer sequences, and product lengths.

Gene target		Primer sequence (5’-3’)	Product length
*Aicda*	forward	CATCCTTTTGCCCTTGTACG	382 bp*
	reverse	CACAGGGTGGGTGTAACAAA	
*Bcl6*	forward	GCGGGAACCACGATCC	237 bp
	reverse	TGCTTTAAAACTGGTGTCCG	
*Ebi3*	forward	TCATTGCCACTTACAGGCTC	427 bp
	reverse	GCTGACACCTGGATGCAA	
*Il10*	forward	TGCCTGCTCTTACTGACTGG	217 bp
	reverse	GGCAACCCAAGTAACCCTTA	
*Irf4*	forward	GGATTGTTCCAGAGGGAGC	277 bp
	reverse	CCTGTCACCTGGCAACC	
*Irf8*	forward	TCTGACCCTCAGGCCTCTT	468 bp
	reverse	GTCACACATCCTGCAATCAGA	
*p35*	forward	TGTGTCTCCCAAGGTCAGC	388 bp
	reverse	GCTCCCTCTTGTTGTGGAAG	
*Prdm1*	forward	GTCGCGGAGACGCAAG	465 bp
	reverse	CCACGCCAATAACCTCTTTG	
*Tgfb1*	forward	ACCAACTATTGCTTCAGCTCC	275 bp
	reverse	TTGCGACCCACGTAGTAGAC	
*Xbp1*	forward	ACACGCTTGGGAATGGACAC	171 bp
	reverse	CCATGGGAAGATGTTCTGGG	

*bp: base pair.

### ELISA

ELISAs were performed following previously published methods [41] and slightly modified where necessary. In brief, blocking, serum dilutions, and the addition of detection antibodies were conducted in 2.5% milk powder in PBS. For anti-Ova antibody measurements, 10 mg/mL (for IgM, IgG1, IgG2b, and IgG2c) or 40 mg/mL (for IgA) of Ova were used to generate Ova-coated ELISA-plates. For the total IgM antibody measurements, an anti-murine IgM antibody from Bethyl Laboratories (Montgomery, USA) was used in a concentration of 5 μg/ml. After incubation with the indicated serum dilutions (1:100 for IgA, IgM, IgG2b, IgG2c; 1:1000 for IgG1) bound antibodies were detected with horseradish peroxidase (HRP)-coupled polyclonal goat anti-mouse IgA-, IgM-, IgG1-, IgG2b-, or IgG2c (the IgG2a haplotype in C57BL/6 mice)-specific antibodies purchased from Bethyl Laboratories (Montgomery, USA). The detection antibodies were diluted according to the manufacturer’s instructions. After incubation with the 3,3´,5,5´-tetramethylbenzidine (TMB) substrate (BD Biosciences, San Diego, USA), the reaction was stopped using the same volume of 4,2% H_2_S0_4_. Eventually, the OD was measured at 450 nm.

### Seahorse mitochondrial stress assay

To analyze mitochondrial respiration of B cells, oxygen consumption rates (OCR) were measured using the Seahorse Mito Stress kit (Agilent, USA). All experiments were performed following an optimized protocol for analyzing lymphocytes previously published elsewhere [42]. B cells were isolated from murine spleens using magnetic activated cell sorting and allowed to rest in *B cell medium* (as described above) for one hour. Negative magnetic activated cell sorting was used because of the simple and fast procedure minimizing the exposure time of the cells to cell buffers and reagents. Additionally, no labeling of the cells of interest is needed, thus minimizing possible effects on cell physiology and metabolism. Subsequently, B cells were cultured for 24 hours in *B cell medium* in the presence or absence of 0.5 mM BA. B cells were then washed and resuspended in Seahorse XF Base Medium (Agilent, USA) supplemented with 10 mM D(+)-Glucose, 1 mM Sodium Pyruvate, and 2 mM L-Glutamine without BA. After counting cells using trypan blue (Thermo Fisher Scientific, Germany), 6 x 10^5^ viable cells per technical replicate were seeded in Seahorse XF96e Cell Culture Microplates (Agilent, USA). After incubation in a CO_2_-free incubator for 60 minutes at 37°C, microplates were transferred to the Seahorse XFe96 Analyzer (Agilent, USA) for measurements. A standard Mito Stress instrument protocol provided by the manufacturer utilizing four modulators of mitochondrial function was applied following the manufacturer’s instructions. Oligomycin A (O, 2 μM), carbonyl cyanide-4-(trifluoromethoxy)phenylhydrazone (FCCP, 2 μM), rotenone (Rot, 1 μM) and antimycin A (AA, 9 μM) were injected at the indicated time points during the assay, enabling the measurement and calculation of several parameters of mitochondrial function. After each injection and at baseline, 3 measurement cycles were conducted, each encompassing 3 min of mixing and 3 min of measuring oxygen consumption rate (OCR) and extracellular acidification rate (ECAR) as surrogates of mitochondrial respiration. 3 biological replicates were included for every experiment with 5–6 technical replicates for every donor mouse.

### Statistical analysis

Sample sizes were determined based on *ex vivo* data and effect sizes from preliminary experiments. The statistical tests employed were selected depending on the number of groups being compared and data distribution. For normally distributed data, either paired or unpaired Student’s t-test was used as applicable. For comparisons of more than two groups, either one-way ANOVA or a mixed-effects model for paired comparisons were employed with Geisser-Greenhouse correction. Tukey’s post-hoc test was applied to follow up on significant results for pairwise comparisons of interest. For non-parametric data sets, the Mann-Whitney U was employed. Statistical analysis was performed using Prism 9.0.0 (GraphPad Software, Inc.). For each graph, data sets from 1–3 representative experiments are shown. If not stated otherwise, experiments were at least replicated twice and are presented as the mean ±SEM. * p < 0.05, ** p < 0.01, and *** p < 0.001.

## Results

### BA induces CD138^high^ PCs and the expression of Prdm1 and Irf4 ex vivo

**Fig 1:**
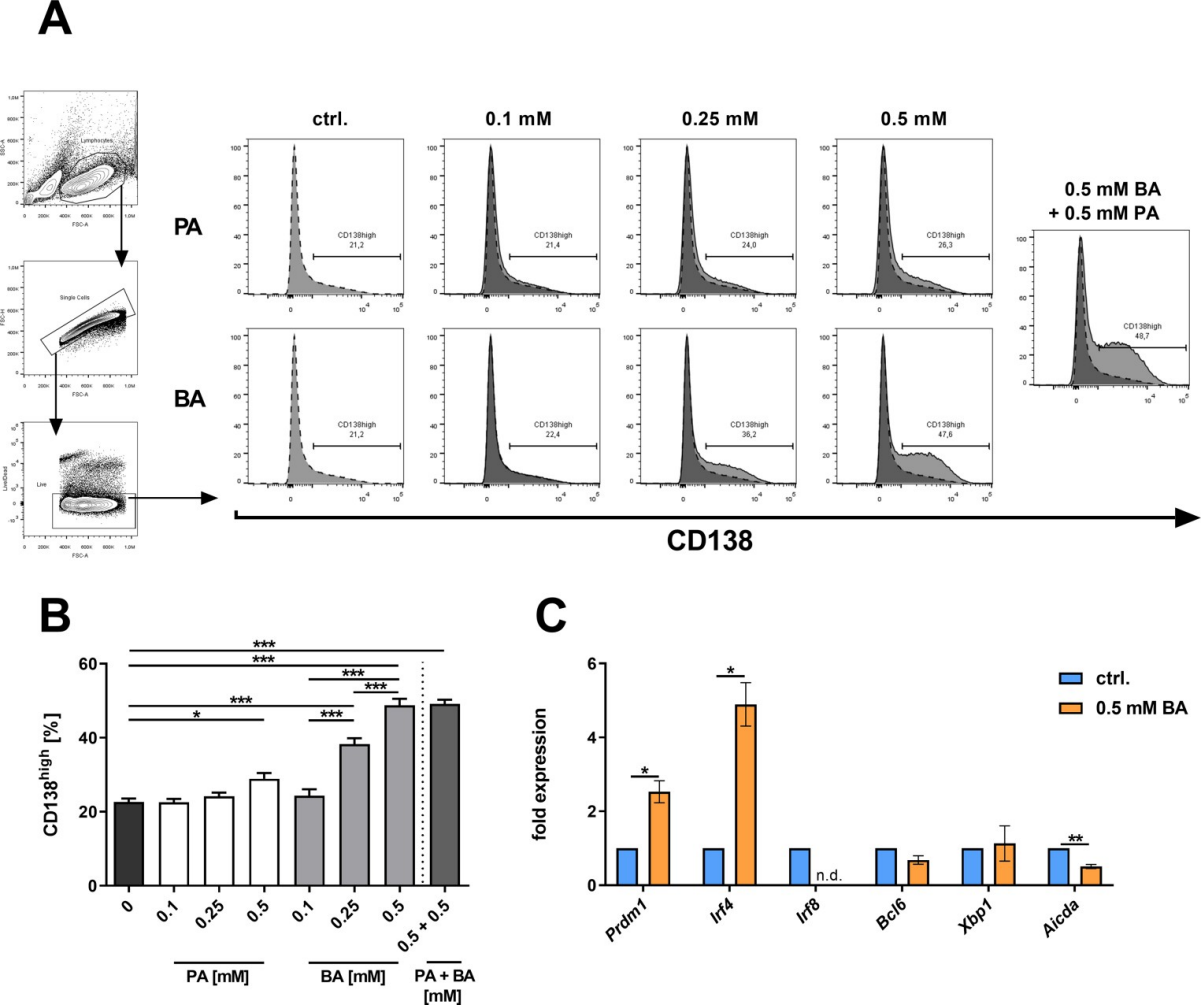
Butyrate promotes the differentiation of CD138^high^ PCs in isolated murine B cells. (A) Flow cytometric gating strategy and representative histogram plots of CD138 expression after treatment of isolated murine splenic B cells with increasing concentrations of PA and/or BA after 4 days of cell culture. (B) Frequencies of CD138^high^ PCs after treatment of isolated murine B cells with the indicated concentrations of PA and/or BA after 4 days of cell culture (n = 5 per group). (C) Gene expression analysis of PC transcription factors in isolated murine splenic B cells after one day of cell culture in the presence of 0.5 mM BA (n = 3). * p < 0.050, ** p < 0.010, *** p < 0.001.

SCFAs increase systemic antibody production in mice invoking potential impact on PC frequencies [9, 39]. To test whether PA and/or BA directly induce differentiation of B cells towards PCs, murine B cells were cultured *ex vivo* in PC-inducing conditions and treated with increasing concentrations of PA or BA for 4 days. The administration of 0.1 or 0.25 mM PA did not change the frequency of induced PCs (defined by CD138^high^ expression). Only 0.5 mM PA showed a slight increase in the frequency of CD138^high^ PCs (p = 0.0215, **Fig 1A and 1B**). In contrast, BA showed a strong dose-dependent effect on the induction of CD138^high^ PCs (**Fig 1A and 1B**). At a concentration of 0.5 mM, BA was sufficient to double the frequency of CD138^high^ PCs (p < 0.0001). The addition of 0.5 mM PA to 0.5 mM of BA did not induce an additional effect on PC differentiation (**Fig 1A and 1B**). Taken together, while PA shows only a slight effect, BA effectively increases CD138^high^ PC differentiation from naïve B cells.

PC differentiation is regulated by several transcription factors including Interferon regulatory factor (Irf) 4, Irf8, B-cell lymphoma 6 protein (Bcl6), X-box binding protein 1 (Xbp1), and PR domain zinc finger protein 1 (Prdm1) [43]. Given the ability of BA to increase frequencies of CD138^high^ PCs, gene expression of transcriptional regulators of PC differentiation was examined in splenic B cells treated with 0.5 mM BA under PC-inducing conditions for one day without additional cell sorting after differentiation. Notably, the expression of *Prdm1*, a master transcriptional regulator of PC differentiation, was upregulated 2.5-fold after incubation with BA (p = 0.0359; **Fig 1C**). Additionally, *Irf4*, an essential inducer of PC differentiation [44], was increased approximately 5-fold (p = 0.0220; **Fig 1C**) and *Irf8*, a potent repressor of PC differentiation and inductor of B cell anergy and non-PC lineages [45], was not detectable in any sample after BA treatment (**Fig 1C**). No changes were found in the gene expression of *Bcl6* and *Xbp1* (**Fig 1C**), which are closely linked to inhibition [46] and induction of PC differentiation [47], respectively. Interestingly, gene expression of *Aicda*, which is crucially involved in antibody class switch recombination in the germinal center [48], was significantly reduced in BA-treated B cell cultures (p = 0.0033; **Fig 1C**). Thus, BA treatment modifies B cell gene expression profiles of important transcriptional regulators towards PC differentiation including *Prdm1* and *Irf4*, while repressing the negative regulator *Irf8* and the class switch inducer *Aicda*.

### BA but not PA induces IL-10 expression in B cells and PCs ex vivo

**Fig 2:**
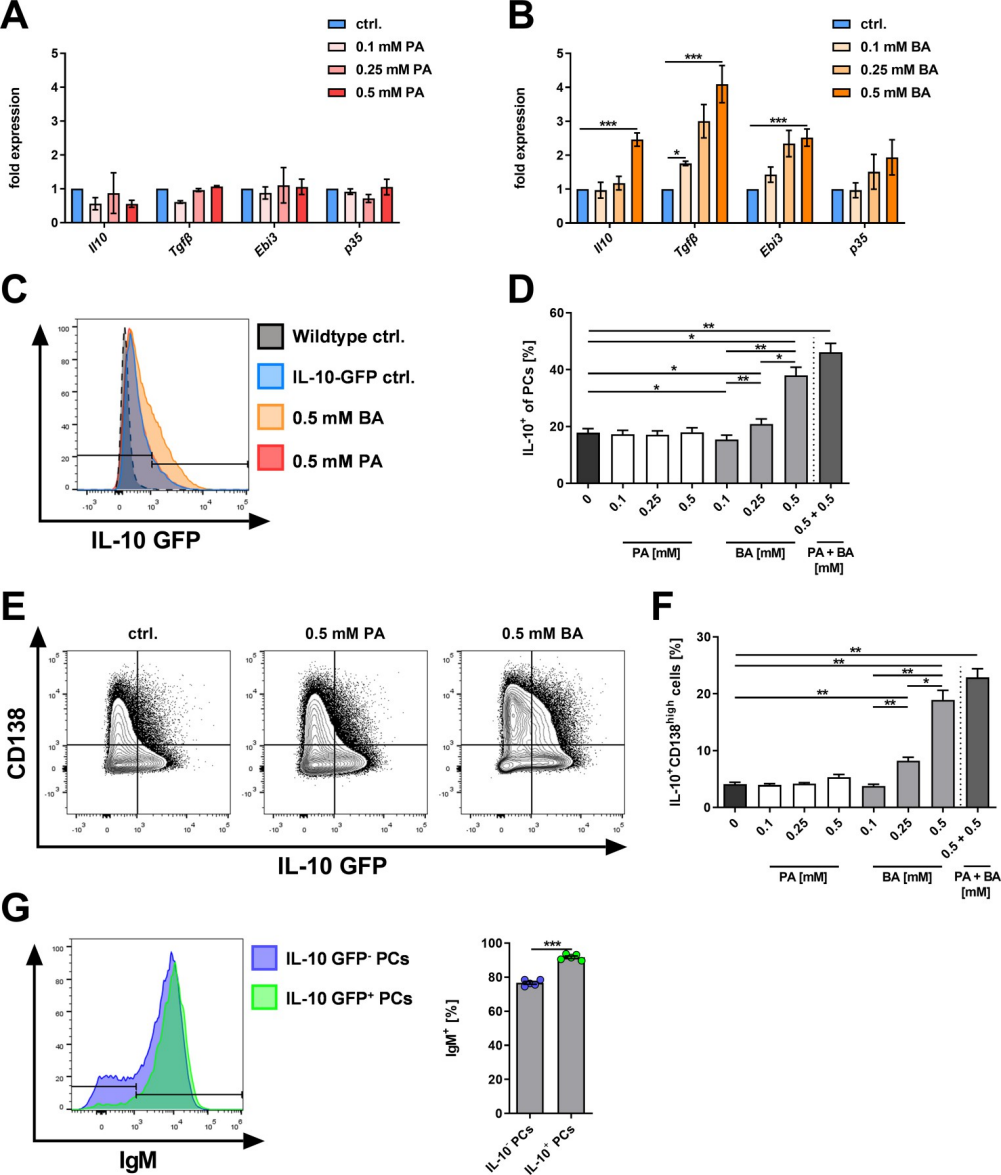
Butyrate promotes the expression of IL-10 in isolated murine B cells. (A, B) Gene expression of anti-inflammatory cytokines after 4 days of B cell culture with increasing concentrations of (A) PA (n = 2–5) or (B) BA (n = 3–14). (C, D) Representative histogram plot and frequencies of IL-10 GFP protein expressing cells among all induced CD138^high^ PCs after 4 days of B cell culture with increasing concentrations of PA and/ or BA (n = 5). (E, F) Representative contour plots and frequencies of IL-10^+^CD138^high^ PCs among all murine B cells after 4 days of B cell culture with increasing concentrations of PA and/or BA (n = 5). (G) Representative histogram plot and frequencies of IgM ^+^ cells among IL-10^+^ and IL-10^-^ CD138^high^ PCs. * p < 0.050, ** p < 0.010, *** p < 0.001.

PCs can induce anti-inflammatory effects by the production of anti-inflammatory cytokines including IL-10, TGFβ, and IL-35 (EBI3/p35). Thus, we next analyzed cytokine gene expression in B cells after four days of treatment with PA and/or BA by qPCR. Treatment with increasing concentrations of PA did not induce *Il10*, *Tgfβ1*, *Ebi3*, or *p35* gene expression (**Fig 2A**). Conversely, treatment of B cells with BA significantly increased gene expression of *Il10* (0.5 mM BA: p = 0.0003), *Tgfβ1* (0.5 mM BA: p = 0.0004), and *Ebi3* (0.5 mM BA: p = 0.0003) in a dose-dependent manner (**Fig 2B**).

To evaluate the effect of BA treatment on IL-10 expression at the protein level, isolated splenic B cells from IL-10-reporter mice were stimulated, and IL-10 expression was evaluated by flow cytometry. After four days of culture, PA treatment did neither influence IL-10 reporter gene expression in all B cells nor CD138^high^ PCs (**Fig 2C–2F**). In contrast, BA treatment increased the frequency of IL-10^+^ cells among all B cells as well as CD138^high^ PCs in a dose-dependent manner (0.5 mM BA: p = 0.0097 for IL-10^+^ cells of all CD138^high^ PCs and p = 0.0016 for IL-10^+^ CD138^high^ PCs of all B cells, **Fig 2C–2F**). When 0.5 mM PA was added to 0.5 mM BA, IL-10 expression in CD138^high^ PCs, as well as the frequency of IL-10^+^ CD138^high^ PCs of all B cells, was only modestly increased compared to 0.5 mM BA (**Fig 2D and 2F**).

To further characterize the IL-10^+^ CD138^high^ PCs present in our B cell culture, the surface expression of IgM was compared between IL-10^-^ and IL-10^+^ CD138^high^ PCs. Strikingly, IL-10^+^ CD138^high^ PCs showed significantly higher expression of IgM on their surface (p <0.0001; **Fig 2G**) indicating that the IL-10^+^ PCs were mainly IgM^+^ PCs. Together these *ex vivo* data suggest that in particular BA, but hardly PA, induce PC differentiation and their expression of anti-inflammatory cytokines including IL-10 at the transcriptional and protein level, indicating the potential of BA to induce regulatory IL-10^+^IgM^+^ PCs.

### BA induces IL-10^+^ CD138^high^ PCs in vivo

Since BA induced CD138^high^ and IL-10^+^ CD138^high^ PCs *ex vivo*, the effects of BA on PC differentiation and IL-10 expression were investigated *in vivo*. BA was administered to mice either enterally via supplementation of drinking water (DW) with 150 mM of BA or systemically via daily intraperitoneal (i.p.) injection (100 mg/kg body weight). No significant changes in body weight or drinking volume were observed during BA treatment (**S1 Fig**). After seven days of BA treatment, mice were immunized with Ovalbumin (Ova)/CFA to induce a systemic immune reaction with increased PC differentiation. We evaluated the frequencies of CD138^high^ PCs and IL-10^+^ CD138^high^ PCs in the spleen of immunized mice treated with BA or vehicle control 12 days post-immunization.

**Fig 3:**
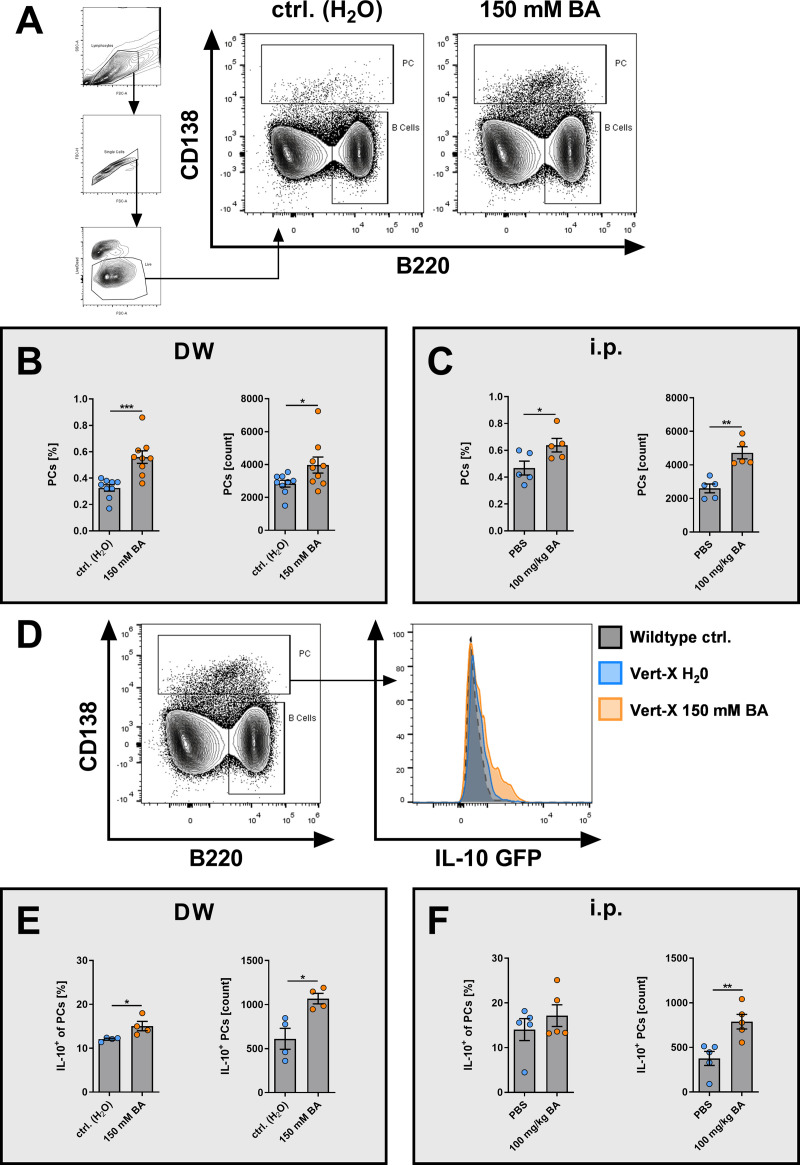
Butyrate promotes the differentiation of CD138^high^ PCs and IL-10^+^ CD138^high^ PCs in Ova-immunized mice. Wildtype or IL-10 reporter mice (IL-10 GFP or Vert-X) were treated for seven days with BA via drinking water or i.p. injection before i.p. immunization with Ova/CFA. 12 days after Ova/CFA treatment splenic cells were harvested and analyzed by flow cytometry. (A) Flow cytometric gating strategy and representative contour plots for CD138^high^ PCs in control and BA-treated mice. (B+C) CD138^high^ PC frequencies and cell counts for mice treated with either (B) drinking water (ctrl.) or BA (150 mM) (n = 9) or (C) daily i.p. injection of either PBS vehicle control or BA (100 mg/kg) (n = 5). (D) Flow cytometric gating strategy and representative histogram plots for IL-10^+^ CD138^high^ PCs in BA-treated IL-10 reporter mice. (E, F) Frequencies and cell counts of IL-10^+^ cells among CD138^high^ PCs for mice treated with either (E) drinking water (ctrl.) or BA (150 mM) (n = 4) or (F) daily i.p. injection of either PBS vehicle control or BA (100 mg/kg) (n = 5). * p < 0.050, ** p < 0.010, *** p < 0.001.

Enteral delivery of BA significantly increased the frequencies (p = 0.0005) and absolute numbers (p = 0.0478) of splenic CD138^high^ PCs compared to control animals (**Fig 3A and 3B**). Similarly, splenic CD138^high^ PC frequencies (p = 0.0461) and absolute numbers (p = 0.0014) were elevated after daily BA i.p. injection compared to the PBS vehicle control (**Fig 3C**), indicating increased differentiation of PCs regardless of the route of administration. Furthermore, enteral BA administration increased the proportion (p = 0.0322) and absolute cell numbers (p = 0.0138) of IL-10^+^ CD138^high^ splenic PCs (**Fig 3D and 3E**). In addition, i.p. administration of BA increased IL-10^+^ CD138^high^ splenic PC numbers (p = 0.0014), whereas the frequency of IL-10^+^ CD138^high^ PCs was only modestly increased in this setting compared to PBS-treated controls (**Fig 3F**). These data demonstrate the ability of BA to induce PCs and their IL-10 expression *in vivo*.

### BA-induced IL-10^+^ CD138^high^ PCs preferentially express IgM in vivo

**Fig 4:**
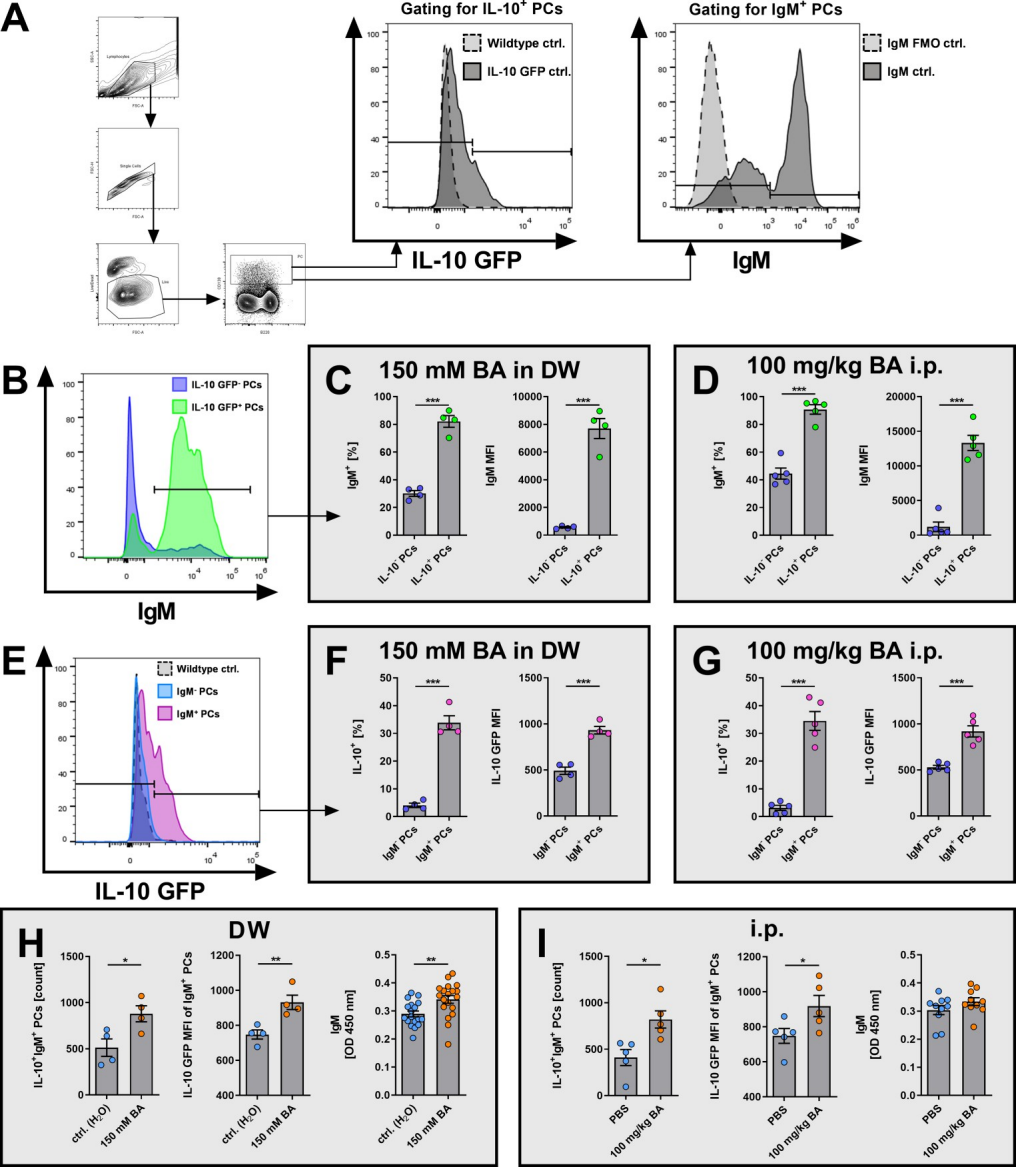
BA induces IL-10^+^CD138^high^ PCs that preferentially express IgM *in vivo*. Wildtype or IL-10 reporter mice (IL-10 GFP) were treated for seven days with BA via drinking water or i.p. injection, before i.p. immunization with Ova/CFA. 12 days after Ova/CFA application splenic cells were harvested and analyzed by flow cytometry. (A) Flow cytometric gating strategy for gating IL-10 GFP^+^ or IgM^+^ PCs in B-I. (B) Representative histogram plot of IgM-expression in IL-10 GFP^+^ and IL-10 GFP^-^ CD138^high^ PCs after treatment with 150 mM BA in DW. (C+D) IgM^+^ PC frequencies and IgM MFI of IL-10 GFP^+^ and IL-10 GFP^-^ CD138^high^ PCs after treatment with (C) 150 mM BA in DW or (D) 100 mg/kg BA i.p. (E) Representative histogram plot of IL-10 GFP-expression in IgM^+^ and IgM^-^ CD138^high^ PCs after treatment with 150 mM BA in DW. (F+G) IL-10 GFP^+^ PC frequencies and IL-10 GFP MFI of IgM^-^ and IgM^+^ CD138^high^ PCs after treatment with (F) 150 mM BA in DW or (G) 100 mg/kg BA i.p. (H+I) Cell counts of IL-10^+^IgM^+^ CD138^high^ PCs, IL-10 GFP MFI of IgM^+^ CD138^high^ PCs, and total serum IgM levels after treatment with (H) 150 mM BA in DW or DW control or (I) 100 mg/kg BA i.p. in PBS or PBS control. OD = optical density. * p < 0.050, ** p < 0.010, *** p < 0.001.

Given the preferential expression of IgM by IL-10^+^ CD138^high^ PCs *ex vivo* (**Fig 2G**), we next examined the expression of IgM in BA-induced IL-10^+^ CD138^high^ PCs *in vivo*. Whereas the main part of IL-10^+^ CD138^high^ PCs expressed IgM (BA in DW: 82.1%; BA i.p. injection: 90.8%), significantly fewer IL-10^-^ CD138^high^ PCs expressed IgM (BA in DW: 30.2%; BA i.p. injection: 44.5%) in BA-treated mice (p < 0.0001 for both treatments; **Fig 4B–4D left panels**). These differences were reflected also by the median fluorescence intensities (MFI) of IgM in IL-10^-^ and IL-10^+^ CD138^high^ PCs (p < 0.0001 for both treatments; **Fig 4C, 4D right panels**). Similarly, the frequency of IL-10^+^ PCs, as well as the IL-10 GFP MFI, were significantly higher among IgM^+^ CD138^high^ PCs compared to IgM^-^ CD138^high^ PCs in BA-treated mice (p < 0.0001 for both treatments; **Fig 4E–4G**).

Furthermore, administration of BA increased the frequency of IL-10^+^IgM^+^ CD138^high^ PCs (DW: p = 0.0290; i.p.: p = 0.0117) and IL-10 GFP MFI in IgM^+^CD138^high^ PCs in comparison to control treatment regardless of the route of administration (DW: p = 0.0093; i.p.: p = 0.0497; **Fig 4H and 4I left and center panels**). Accordingly, treatment with BA via drinking water induced a significant increase of total serum IgM levels (p = 0.0057, **Fig 4H right panel**), while i.p. injection of BA slightly, but not significantly increased serum IgM levels (p = 0.1796, **Fig 4I right panel**). These data indicate that BA-induced IL-10^+^ CD138^high^ PCs preferentially express IgM *in vivo*.

### BA induces anti-Ova IgM but inhibits class switching to pathogenic anti-Ova IgG2b upon Ova/CFA immunization

**Fig 5:**
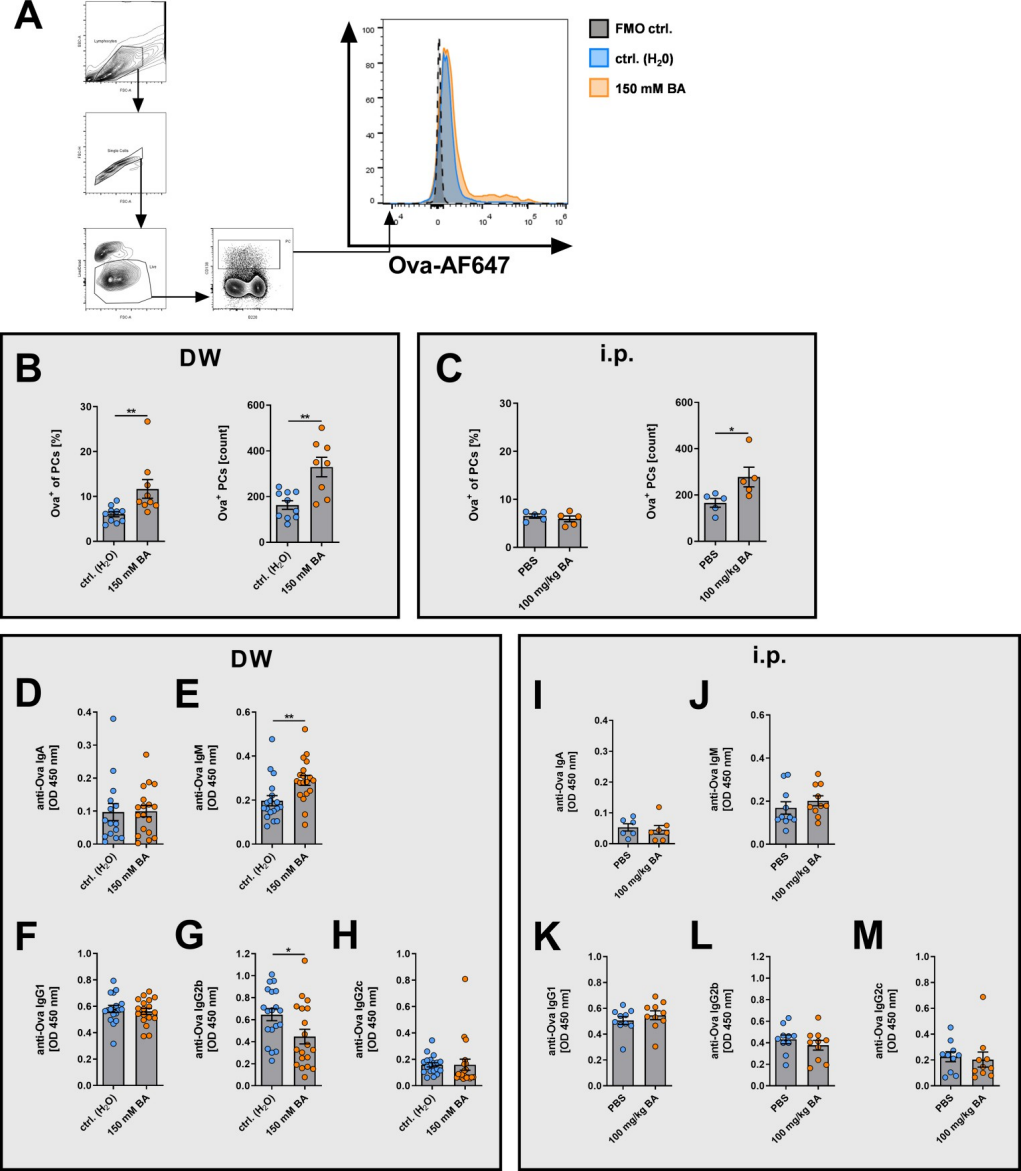
BA in drinking water increases Ova-specific PCs and serum levels of anti-Ova IgM, but blocks class switching to anti-Ova IgG2b. Mice were treated for seven days with BA via drinking water or i.p. injection, before i.p. immunization with Ova/CFA. 12 days after Ova/CFA application splenic cells were harvested and analyzed by flow cytometry. (A) Flow cytometric gating strategy and representative histogram plots for staining of Ova-specific PCs in BA-treated mice (drinking water). (B, C) Ova-specific PC frequencies and cell counts of mice treated with either (B) water or BA (150 mM) or (C) PBS vehicle control or BA (100 mg/kg) by i.p. injection. (D-M) Serum levels of anti-Ova IgA (D, I), IgM (E, J), IgG1 (F, K), IgG2b (G, L), and (H, M) IgG2c antibodies for mice treated with either (D-H) water or BA (150 mM) or (I-M) PBS vehicle control or BA (100 mg/kg) by i.p. injection. OD = optical density. * p < 0.050, ** p < 0.010.

Next, we analyzed the influence of BA on the Ova-specific B cell response upon Ova/CFA immunization. To identify Ova-specific CD138^high^ PCs 12 days after Ova/CFA immunization, cells were additionally stained extra- and intracellularly with fluorophore-conjugated Ova. Enteral administration of BA increased the frequency of Ova-specific PCs among all CD138^high^ PCs (p = 0.0139), as well as the absolute number of Ova-specific CD138^high^ PCs (p = 0.0389, **Fig 5A and 5B**), while i.p. administration of BA did not increase the frequency, but the absolute number of Ova-specific PCs (p = 0.0436, **Fig 5C**). Serum levels of anti-Ova IgA (**Fig 5D**) and anti-Ova IgG1 levels (**Fig 5F**) remained unchanged after the application of 150 mM BA in drinking water. However, anti-Ova IgM levels were significantly elevated (p = 0.0069, **Fig 5E**). Moreover, anti-Ova IgG2b (p = 0.0270, **Fig 5G**) and in tendency anti-Ova IgG2c (**Fig 5H**) levels were reduced after administration of 150 mM BA in drinking water. These differences were also found in tendency, but without statistical significance after i.p. injection of BA (**Fig 5I–5M**). Thus, the induction of IL-10^+^IgM^+^ CD138^+^ PCs after BA treatment was associated with increased systemic anti-Ova IgM levels and inhibition of class switching of antigen-specific antibodies to the pathogenic IgG2b subclass particularly after enteral application of BA.

### Specific inhibition of HDAC3 is sufficient to induce IL-10^+^ PCs

Next, we investigated the effector mechanisms of BA. GPR43/FFAR2 activation is an underlying mechanism for the immunomodulatory effects of BA on T cells and neutrophils [3]. In contrast to other SCFA-receptors (Gpr41, Gpr109a, or OLFR78), GPR43 is expressed also on activated murine B cells [9]. Thus, we next investigated whether GPR43 activation might be involved in BA-mediated PC induction. Treatment of isolated murine B cells with 0.5 mM BA increased CD138^high^ PCs (p < 0.0001; **S2A and S2B Fig**), whereas the specific GPR43 agonist did not induce similar changes (p = 0.5997 vs. control, p < 0.0001 vs. BA; **S2A and S2B Fig**), even when used in a concentration of 1 μM exceeding the reported IC50 value (IC50 = 0.7 μM [49]). These data suggest that GPR43 activation is not sufficient to induce PC differentiation similar to BA in murine B cells.

**Fig 6:**
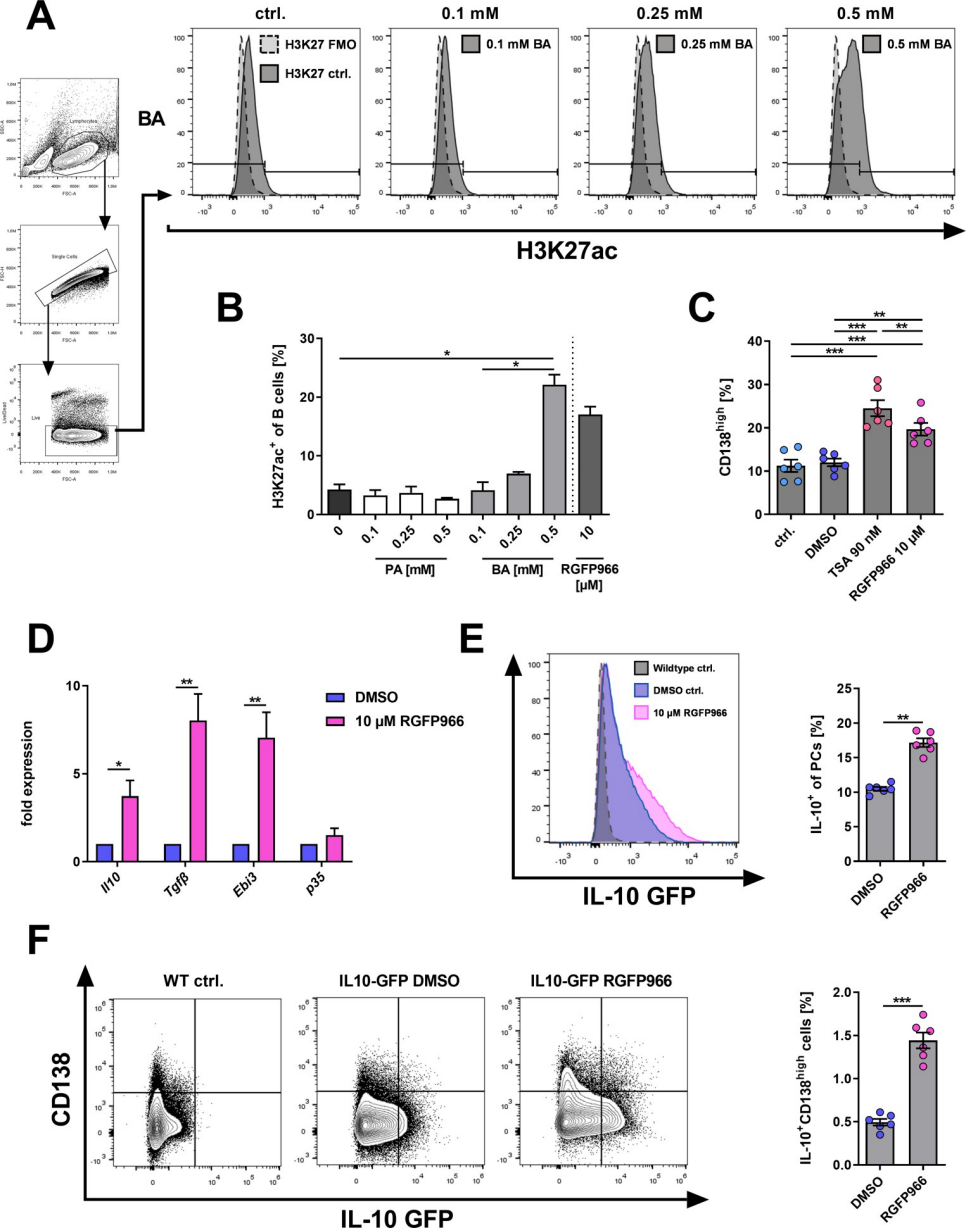
Specific HDAC3 inhibition by RGFP966 promotes the differentiation of CD138^high^ PCs, IL-10^+^ B cells, and IL-10^+^ CD138^high^ PCs in isolated murine B cells. (A) Flow cytometric gating strategy and representative histogram plots of H3K27 acetylation in isolated murine B cells after 3 days of cell culture. (B) Frequencies of H3K27ac^+^ cells in isolated murine B cells after 3 days of incubation with increasing concentrations of PA and BA (n = 3). Treatment with RGFP966 (10 μM) as a positive control. (C) Frequencies of CD138^high^ PCs after 4 days of incubation of isolated murine B cells with HDAC inhibitor TSA or HDAC3-specific inhibitor RGFP966. (D) Gene expression of anti-inflammatory cytokines after 4 days of cell culture with 10 μM RGFP966 (n = 6). (E) Representative overlay histogram plot and IL-10 GFP protein expression among all CD138^high^ PCs after 4 days of B cell culture with 10 μM RGFP966 (n = 6). (F) Representative contour plots and frequencies of IL-10^+^ CD138^high^ PCs among all B cells after 4 days of B cell culture with 10 μM RGFP966. TSA = Trichostatin A. * p < 0.050, ** p < 0.010, *** p < 0.001.

As immunomodulatory functions of BA are also linked to the inhibition of class I and II HDACs [3, 50], the effects of BA on histone acetylation in isolated murine B cells were analyzed using a fluorophore-conjugated antibody against the acetylated lysine residue at N-terminal position 27 of the histone protein H3 (H3K27ac). Increasing concentrations of PA did not affect H3K27 acetylation, while 0.5 mM BA significantly increased the frequency of H3K27ac^+^-stained B cells from 4% to 22% (p = 0.0274) similar to the effects of HDAC3 inhibitor RGFP966 (**Fig 6A and 6B**), indicating HDAC-inhibitory properties of BA on murine B cells.

Considering the HDAC-inhibitory capacity of BA, we examined next whether HDAC inhibition recapitulates the induction of IL-10^+^ PCs by BA. Employing HDAC-specific inhibitors we examined whether HDAC inhibition is sufficient to induce regulatory PCs under the same conditions as BA *ex vivo*. Indeed, incubation of isolated splenic B cells with Trichostatin A (TSA), a chemical inhibitor of class I and II HDACs, was sufficient to recapitulate the induction of CD138^high^ PCs by BA (p = 0.0007; **Fig 6C**). Among all HDAC isotypes, HDAC3 inhibition is suggested to be specifically responsible for immunomodulatory effects of BA, but not PA [51, 52]. Therefore, to examine the sufficiency of HDAC3 inhibition on inducing PC differentiation isolated murine B cells were treated with the specific HDAC3-inhibitor RGFP966. Notably, RGFP966 treatment was sufficient to induce CD138^high^ PCs (**Fig 6C**). Together, these data indicate that HDAC3 inhibition plays an important role in the induction of CD138^high^ PCs.

To examine whether HDAC3 inhibition was furthermore sufficient to recapitulate the induction of the anti-inflammatory cytokines *Il10*, *Tgfβ1*, and *Il35* by BA, gene expression levels after treatment of B cells with RGFP966 for four days were determined by qPCR. Indeed, while p35 expression was not changed significantly, the expression levels of *Il10* (p = 0.0284), *Tgfb1* (p = 0.0055), and *Ebi3* (p = 0.0087) were increased significantly (**Fig 6D**). Moreover, RGFP966 treatment increased the frequencies of IL-10^+^ cells among all PCs (p = 0.0007) and IL-10^+^ CD138^high^ PCs among all B cells (p < 0.0001) as shown with isolated B cells from IL-10-reporter mice (**Fig 6E and 6F**) recapitulating the effects observed from BA treatment. Combined, these results indicate that HDAC3 inhibition but not GPR43 activation is sufficient to induce CD138^high^ PCs, the expression of anti-inflammatory cytokines, and the induction of IL-10^+^ PCs in isolated B cells similar to the effects observed for BA.

### BA decreases mitochondrial metabolism and respiration in B cells

**Fig 7:**
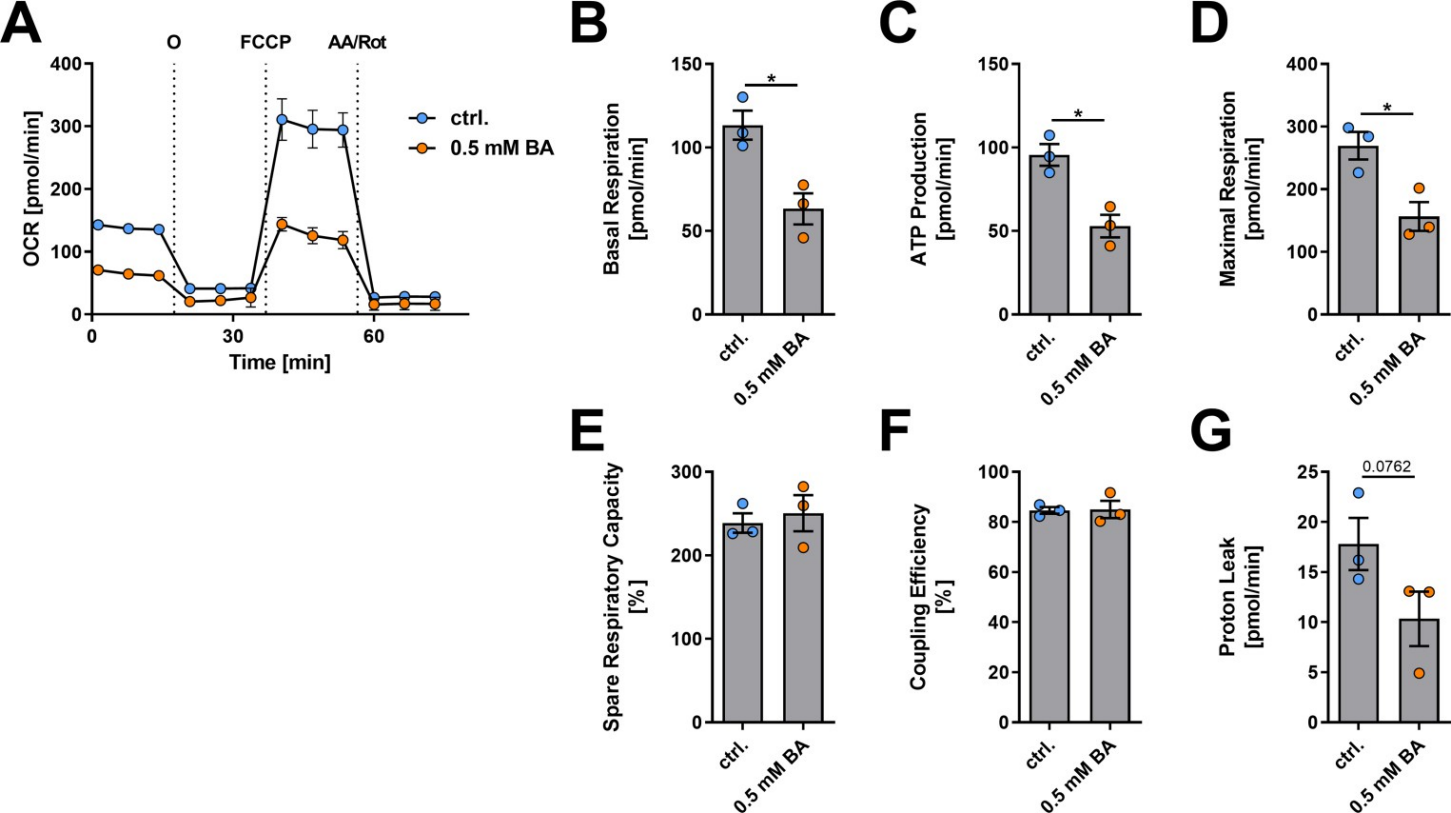
Seahorse analysis of BA effects on mitochondrial metabolism in isolated murine B cells. (A) OCR measurements of isolated murine B cells in the Mito-Stress assay after one day of incubation with 0.5 mM BA. The following parameters were calculated (n = 3) from the initial measurements of the oxygen consumption rates at baseline and after injection of oligomycin (O), FCCP, and antimycin A/rotenone (AA/Rot): (B) Basal respiration, (C) ATP production, (D) maximal respiration, (E) spare respiratory capacity in %, (F) coupling efficiency in %, and (G) proton leak. * p < 0.050.

BA modulates mitochondrial metabolism in various tissues including skeletal muscle [53], brown adipose tissue [53], liver [54], and colon [55]. Mitochondrial function is central in regulating PC differentiation [56]. Thus, we next examined the impact of BA treatment on B cell mitochondrial metabolism by conducting mitochondrial stress assays. Isolated splenic B cells were subjected to BA treatment for one day (0.5 mM) and mitochondrial respiration was measured using Seahorse Extracellular Flux Analysis (Agilent, USA) (**Fig 7A**). In short, extracellular flux analysis allows for the measurement of oxygen consumption rate (OCR) as a surrogate of mitochondrial metabolism. During the assay, the mitochondrial chain is modified to enable the measurement of several parameters of mitochondrial respiration, including ATP production, proton leak, spare respiratory capacity, as well as baseline and maximal respiration.

At baseline, the OCR representing basal respiration in isolated B cells decreased from 113.4 to 63.3 pmol/min after treatment with 0.5 mM BA (p = 0.0170; **Fig 7B**). Accordingly, ATP-production (p = 0.0105; **Fig 7C**) was significantly reduced. Similarly, maximal respiration after uncoupling of the respiratory chain by FCCP (p = 0.0235; **Fig 7D**) was decreased, whereas no significant differences were found for spare respiratory capacity, coupling efficiency, and proton leak (**Fig 7E–7G**). Together, these data suggest that mitochondrial metabolism of isolated B cells, which is deeply involved in deciding B cell fate [56], is significantly inhibited after 1 day of incubation with 0.5 mM BA.

### BA and specific HDAC3 inhibition decrease mitochondrial superoxide levels to induce PCs

**Fig 8:**
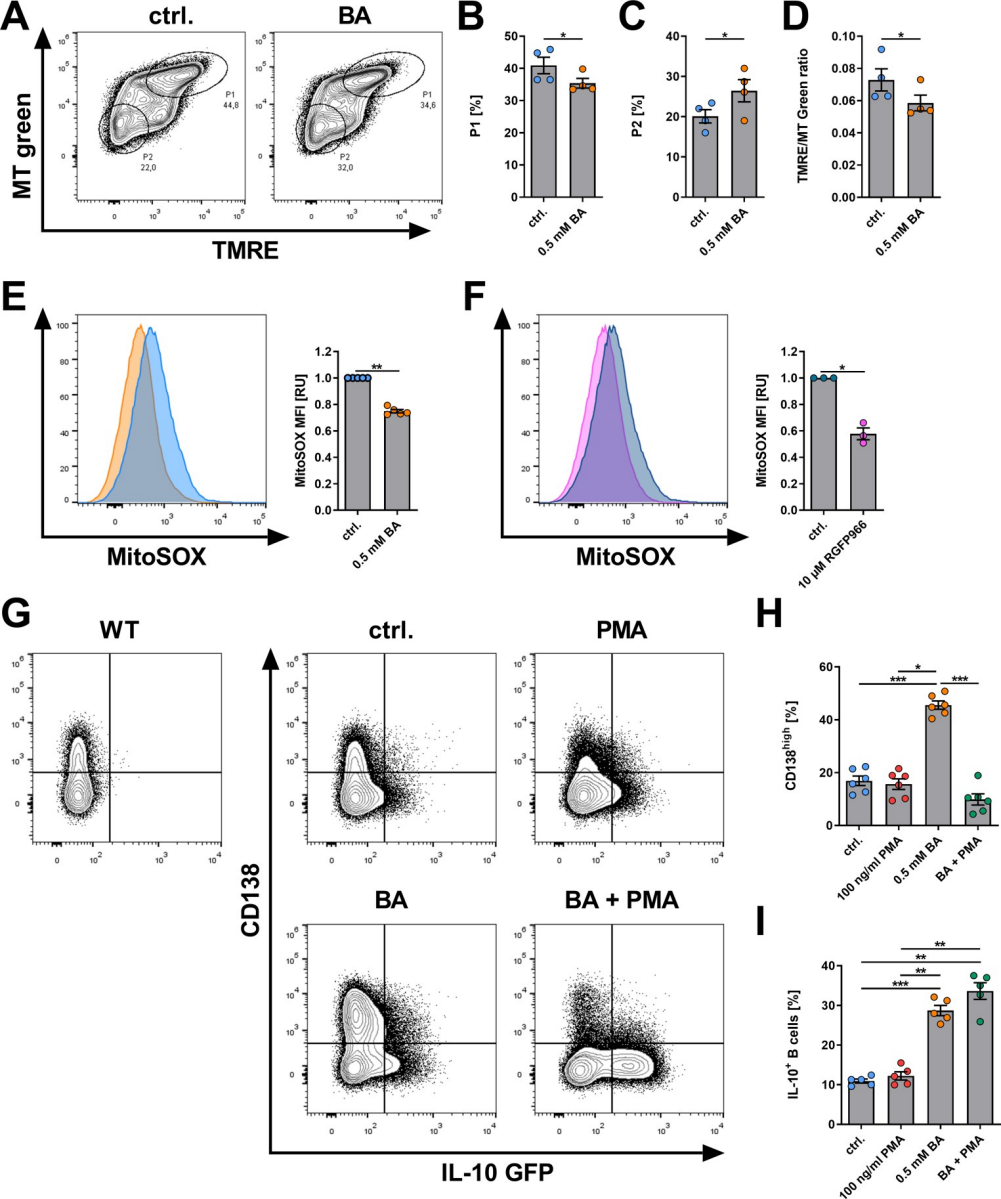
BA decreases mitochondrial membrane potential and superoxide production after one day of cell culture. (A) Representative contour plots of MT-Green/TMRE-stained B cells after incubation with 0.5 mM BA for 1 day. (B) P1 frequencies after incubation with 0.5 mM BA for 1 day indicating MT-Green^high^ TMRE^high^ B cells (n = 4). (C) P2 frequencies after incubation with 0.5 mM BA for 1 day indicating MT-Green^low^ TMRE^low^ B cells (n = 4). (D) Ratio of TMRE MFI and Mito-Green MFI after incubation with 0.5 mM BA for 1 day indicating MT-Green^low^ TMRE^low^ B cells (n = 4). (E) Incubation with 0.5 mM BA for 1 day decreased the levels of mitochondrial superoxide levels indicated by MitoSOX staining (n = 5). (F) Incubation with 10 μM RGFP966 for 1 day decreased the levels of mitochondrial superoxide levels indicated by MitoSOX staining (n = 3). (G) Representative contour plots of CD138 and IL-10 GFP expression in isolated splenic B cells from IL-10 reporter mice after treatment with 0.5 mM BA and/or 100ng/ml PMA. (H) Frequencies of CD138^high^ PCs after treatment of isolated murine B cells with 0.5 mM BA and/or 100ng/ml PMA after 4 days of cell culture (n = 6). (I) Frequencies of IL-10^+^ B cells after treatment of isolated murine B cells with 0.5 mM BA and/or 100ng/ml PMA after 4 days of cell culture (n = 5). PMA = Phorbol-12-myristate-13-acetate. * p < 0.050, ** p < 0.010, *** p < 0.001.

Mitochondrial respiration is tightly linked to the production of reactive oxygen species (ROS), especially superoxide. Notably, low mitochondrial respiration predetermines B cell differentiation towards PCs [56]. Thus, we next analyzed the effects of BA on mitochondrial activity and superoxide production by flow cytometry. A combination of fluorescent stains specific for mitochondrial mass (MT Green) and mitochondrial membrane potential (TMRE) was used to evaluate mitochondrial activity. One day of BA treatment decreased the proportion of the P1 population representing MT^high^TMRE^high^ B cells with highly active mitochondria from 40.9 to 35.4% (p = 0.0497, **Fig 8A and 8B**), while the P2 population representing MT^low^TMRE^low^ B cells with less mitochondrial activity increased from 20.1 to 26.5% (p = 0.0232, **Fig 8A and 8C**). The MFI ratio of TMRE to MT green, which is a parameter used to determine the activity of mitochondria in relation to total mitochondrial mass, decreased with BA treatment (p = 0.0147, **Fig 8D**). Together these data indicate that a decrease in active mitochondrial mass correlates with the mitochondrial respiratory capacity following BA treatment (**Fig 7A–7G**).

Next, specific staining for mitochondrial superoxide (MitoSOX) was used as a surrogate for mitochondrial superoxide levels given their inhibitory influence on PC differentiation [56]. BA treatment reduced MitoSOX MFI by 25% (p < 0.0001, **Fig 8E**). Furthermore, this effect was recapitulated by a 42% decrease of MFI after specific inhibition of HDAC3 by RGFP966 (p = 0.0110, **Fig 8F**), indicating a reduction of mitochondrial superoxide levels by BA.

To investigate whether the reduction in superoxide levels is merely a byproduct or indeed necessary for BA effects on PC and IL-10 induction, PMA, a well-established inducer of mitochondrial superoxide [57–59], was used. PMA alone did not significantly change the proportion of CD138^high^ PCs compared to the control. However, PMA completely abrogated BA-induced PC differentiation (BA vs. BA+PMA: p = 0.0004; **Fig 8G and 8H**), whereas BA-induced IL-10 expression was not significantly affected by PMA (**Fig 8G and 8I**). Similar results were also obtained when 2DG was used as an inducer of mitochondrial ROS [56, 60, 61] and RGFP966 for inhibition of HDAC3 (**S3 Fig**). In summary, BA reduction of mitochondrial activity and superoxide production may act as a key factor in determining B cell differentiation towards PCs. Accordingly, abolished induction of PCs by BA after treatment with PMA or 2DG indicates that reduced superoxide levels are likely necessary for BA-induced PC differentiation, but not for the induction of IL-10.

## Discussion

Here, we show that BA enhances the differentiation of IgM^+^ CD138^high^ PCs, IL-10 expression, and IL-10^+^IgM^+^ CD138^high^ PCs. *Ex vivo* studies indicated that specific HDAC3 inhibition was sufficient to elicit both effects similar to BA, whereas reduced mitochondrial superoxide levels following HDAC3 inhibition were necessary for PC differentiation, but not IL-10 expression. The crucial relevance of HDAC3 for PC differentiation is underlined by only modest effects when PA was used. Although PA is a similarly potent HDAC inhibitor, it specifically does not inhibit the function of HDAC3 [51]. The application of a specific inhibitor of HDAC3, or BA, to induce regulatory IL-10^+^IgM^+^ PCs might therefore represent an interesting opportunity for novel treatment options and could be investigated in models of B cell-dependent inflammatory autoimmune diseases [29], e.g. models of rheumatoid arthritis [62], systemic lupus erythematosus [62], or multiple sclerosis [63].

In isolated murine B cells and Ova/CFA-immunized mice, BA promoted the differentiation of CD138^high^ PCs. Notably, enteral or systemic administration of BA only 7 days before Ova/CFA immunization efficiently induced the observed effects, indicating a short-term BA application to be sufficient to induce changes in PC differentiation. Supporting evidence stems from a previous study demonstrating that SCFA administration via a high-fiber diet or drinking water supports host IgA and IgG antibody responses, also demonstrating increased PC differentiation [39]. Inhibition of HDACs by SCFAs may provide an underlying mechanism in their studies [39]. These results were further corroborated by *Sanchez et al*. showing increased PC differentiation in low doses of SCFAs in drinking water [9]. However, opposing effects with reduced PC differentiation were observed when a highly concentrated mixture of SCFAs and butyrate prodrugs (140mM BA and 150mM PA, 20 mg/ml tributyrin emulsion) was administered [9]. Taking into account that the different SCFAs might/can induce distinct mechanisms, mixtures of different SCFAs might result in different effects possibly explaining discrepant observations in such studies. Furthermore, recent studies with dietary fiber have shown that certain gut bacteria can generate BA, whereas other gut bacteria primarily generate the SCFAs PA or acetate (C2) [3–5]. Different compositions of the microbiome might therefore generate distinct SCFA proportions with distinct influences. Shifting the gut microbiome to BA-generating species might therefore be an attractive approach to induce regulatory IL-10^+^ PCs for the treatment of inflammatory immune diseases.

Since regulatory CD138^high^ PCs have been identified as the major source of B cell-derived IL-10 and IL-35 [64–67] we analyzed the effects of BA on the expression of anti-inflammatory cytokines in PCs. BA increased the expression of such cytokines (e.g., IL-10) *ex* and *in vivo*. Supporting data stems from a recent, well-executed study by *Luu et al*., reporting that BA, as well as the less studied SCFA pentanoate (C5), were able to effectively induce the expression of IL-10 in B cells and that pentanoate-treated B cells were able to suppress autoimmunity in a murine model of multiple sclerosis [25]. In this publication, however, the possible expression of additional Breg and PC markers and the secretion of antibodies have not been investigated, allowing for speculation, whether the induced cells might in fact be regulatory IL-10^+^ PCs [25]. Furthermore, BA induced IL-10^+^ Bregs in a model of Sjögren’s Syndrome effectively ameliorating the disease, highlighting the potential therapeutic implications of inducing Bregs in autoimmune diseases [27].

Further characterization of BA-induced PCs revealed that approximately 90% of the splenic IL-10^+^ PCs expressed IgM *in vivo*. Similarly, IL-10 expression was observed in significantly higher frequencies in IgM^+^ PCs. These results are corroborated by previous literature reporting Breg subsets to frequently express IgM [68]. Most notably, anti-inflammatory IL-10 and IL-35-producing PCs, which are the main source of these cytokines in models of infection and autoimmunity, expressed IgM at exceptionally high levels [65]. The number of splenic IL-10^+^IgM^+^ PCs and the expression of IL-10 in splenic IgM^+^ PCs as determined by fluorescence intensity of IL-10 GFP were significantly increased after the administration of BA by drinking water or i.p. injection, underlining the capacity of BA to induce IgM^+^ PCs of a regulatory phenotype. Accordingly, BA increased the total serum IgM levels in our studies.

By further investigating the anti-inflammatory effects of BA, we identified that the BA-induced IL-10^+^IgM^+^ PCs were associated with the generation of more Ova-specific PCs and anti-Ova IgM serum levels in Ova/CFA-immunized mice. Furthermore, class switching to anti-Ova IgG2b and in tendency IgG2c was decreased after BA treatment via drinking water, matching reduced *Aicda* expression *in vitro*. Notably, murine IgG2b and IgG2c are the two IgG subclasses that are particularly associated with proinflammatory properties, such as activation of the complement system [37], and various immune cells via high affinity-binding to activating Fcγ receptors [36, 37, 69, 70]. BA-induced inhibition of switching to antigen-specific IgG subclasses might be an IL-10-dependent second pathway of BA to inhibit inflammatory immune responses.

The role of BA as an inhibitor of HDACs in various cell types including B cells is well established [25, 71]. Here, we demonstrated increased histone (H3K27) acetylation as a surrogate for HDAC inhibition. Similarly, HDAC3 knockout in murine T cells leads to increased H3K27 acetylation and, notably, *Prdm1* transcription [72]. Additionally, HDAC3 inhibition has been shown to be the culprit of the immunomodulatory effect of BA on macrophages [52], while H3K27 acetylation is associated with CD138 expression during PC differentiation [73]. Accordingly, the unspecific HDAC inhibitor TSA and the HDAC3 inhibitor RGFP966 recapitulated the effects of BA on regulatory PC differentiation from isolated murine B cells *ex vivo*. Gene expression of master regulators of PC differentiation *Prdm1* and *IRF4* were increased in isolated murine B cells after one day of culture with BA, while expression of the negative regulator *IRF8* was not detectable. In contrast to BA, PA was not an effective inducer of IL-10^+^ PC differentiation or H3K27 acetylation and is ineffective as an HDAC3 inhibitor as well [51, 52] further supporting a central role of HDAC3 inhibition for the observed effects.

Mitochondrial ROS production plays a significant role in the regulation of stem cell differentiation [74] and reduced ROS levels favor PC differentiation from activated B cells by increasing *Prdm1* expression [56]. BA-inhibited mitochondrial metabolism and superoxide production in B cells likely contribute to the increased PC differentiation observed here. Similarly, HDAC3 inhibition by RGFP966 reduced mitochondrial superoxide levels. Accordingly, ROS production has been recently shown to decrease in the absence of HDAC3 in macrophages [75] linking HDAC3 inhibition by BA and reduced ROS levels. The abrogation of BA-induced PC differentiation by the mitochondrial superoxide inducers PMA [57–59] and 2DG [56] further indicates that decreased mitochondrial superoxide levels are necessary for BA-induced PC differentiation. However, the proposed underlying mechanisms are based mainly on *ex vivo* data and need verification in additional *in vivo* studies. Interestingly, PMA and 2DG did not abrogate the expression of IL-10 in B cells similarly to PC differentiation. Thus, reduced mitochondrial superoxide levels seem to be necessary for the induction of PCs by BA, whereas IL-10 expression is likely induced independently from mitochondrial superoxide levels. It might therefore be possible that BA-induced reduction of mitochondrial superoxide levels induces regular PCs and that another unknown anti-HDAC3-dependent mechanism induced by BA elicits the anti-inflammatory phenotype including IL-10 expression.

In this study, BA induced the differentiation of IL-10^+^IgM^+^ PCs in short-term experiments in adult mice, most likely due to HDAC-related epigenetic mechanisms (i.e., increased histone acetylation). Previous literature has suggested that epigenetic changes induced by microbially-derived BA in early life lead to long-term protection against allergic diseases [76–79]. These effects have mainly been attributed to the correction of the exacerbated Th2-response observed in allergy [79, 80]. However, regulatory B cells play a crucial role in protecting against allergic diseases [81], highlighting the relevance of future studies on BA-mediated induction of regulatory PCs in early life and long-term experiments. Possibly, induction of IL-10^+^ PCs by BA in early life might be an effective way to protect against allergy and autoimmunity, especially in the long run.

In conclusion, we showed that the microbial metabolite BA induces the differentiation of regulatory IL-10^+^IgM^+^ PCs that was associated with a reduced class switch to antigen-specific pathogenic murine IgG2 antibodies. Contributing mechanisms to the induction of regulatory IL-10^+^IgM^+^ PCs likely encompass the downregulation of HDAC3 activity. Additionally, reduced mitochondrial superoxide levels after BA treatment are necessary for PC differentiation, but not IL-10 induction. Thus, epigenetic modulation of PC differentiation towards a more regulatory phenotype by BA or targeted inhibition of HDAC3 might be a potential therapeutic target in autoimmune and allergic diseases.

## Supporting information

S1 Fig Butyrate treatment does not affect weight development and drinking volume after Ova/CFA immunization. (A) Weight development of mice treated with daily intraperitoneal injections of BA (100 mg/kg) after Ova/CFA immunization (n = 5). (B) Weight development of mice treated with 150 mM BA in drinking water *ad libitum* after Ova/CFA immunization (n = 10). (C) Average daily drinking volume of mice treated with 150 mM BA in drinking water *ad libitum* after Ova/CFA immunization (n = 10).
(TIF)

S2 Fig GPR43/FFAR2 agonist does not induce CD138^high^ PCs in isolated murine B cells. (A) Flow cytometric gating strategy and representative histogram plots for CD138^high^ PCs after treatment with 0.5 mM BA or 1 μM allosteric GPR43 agonist. (B) PC frequencies after treatment of isolated murine B cells with 0.5 mM BA or 1 μM allosteric GPR43 agonist for 4 days of cell culture. ** p < 0.010, *** p < 0.001.
(TIF)

S3 Fig 2-DG abrogates the induction of CD138^high^ PCs by BA and RGFP966 in isolated murine B cells. (A) Representative contour plots of CD138 and IL-10 GFP expression in isolated splenic B cells from IL-10 reporter mice after treatment with 0.5 mM BA and 200 μM 2DG. (B) Frequencies of CD138^high^ PCs after treatment of isolated murine B cells with 0.5 mM BA and/or 200 μM 2DG after 4 days of cell culture (n = 5). (C) Frequencies of IL-10^+^ B cells after treatment of isolated murine B cells with 0.5 mM BA and/or 200 μM 2DG after 4 days of cell culture (n = 6). (D) Representative histogram plots of CD138 expression in isolated splenic B cells after treatment with 10 μM RGFP966 and/or 200 μM 2DG. (E) Frequencies of CD138^high^ B cells after treatment of isolated murine B cells 10 μM RGFP966 and/or 200 μM 2DG after 4 days of cell culture (n = 6). * p < 0.050, ** p < 0.010, *** p < 0.001.
(TIF)
