# Supplementary figures and images for: Microbial metabolite butyrate promotes induction of IL-10+IgM+ plasma cells
Source: PLoS One. 2022 Mar 25;17(3):e0266071. doi: 10.1371/journal.pone.0266071 (PMC8956175; doi:10.1371/journal.pone.0266071)

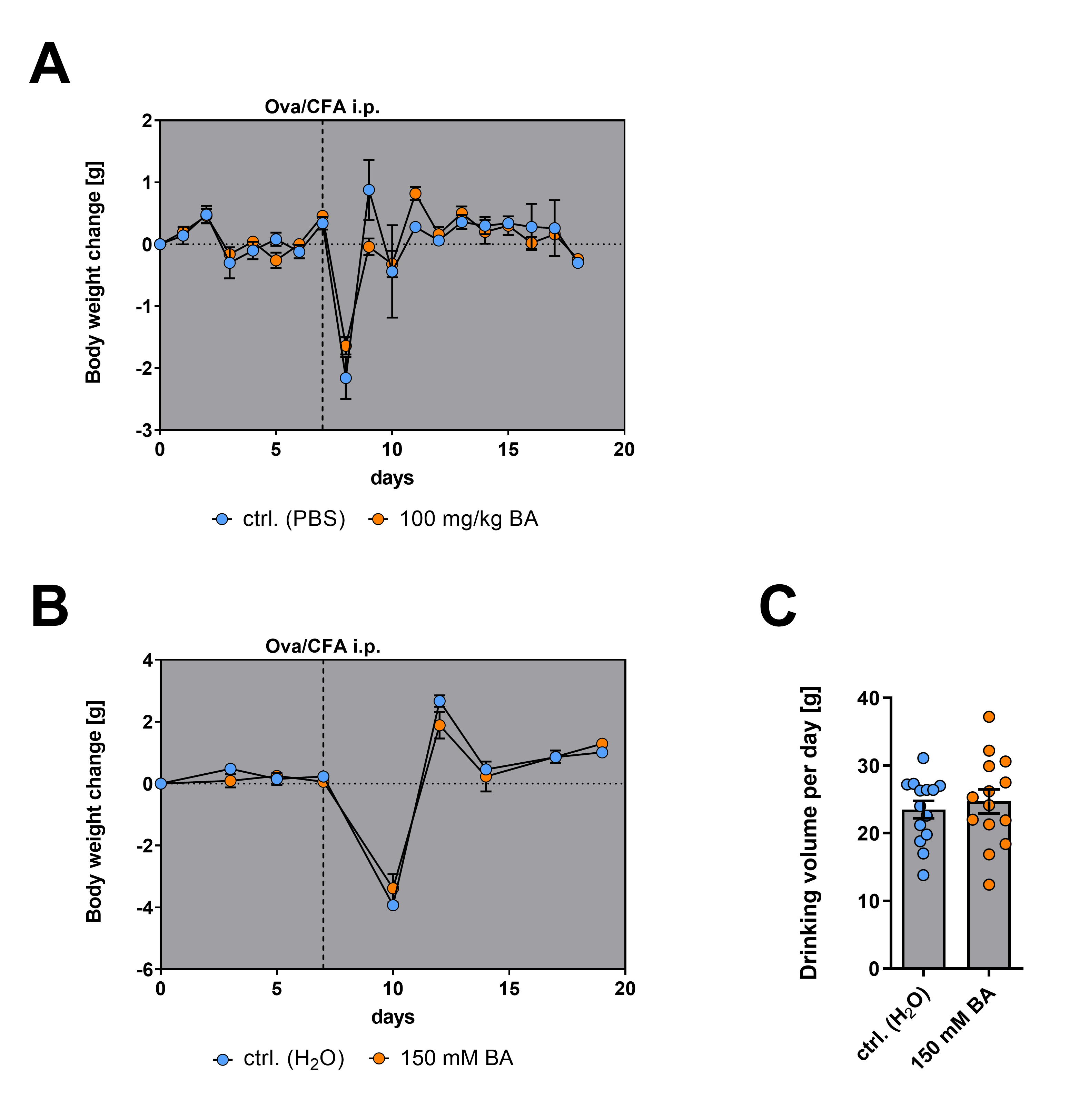

Supplement: S1 Fig — (A) Weight development of mice treated with daily intraperitoneal injections of BA (100 mg/kg) after Ova/CFA immunization (n = 5). (B) Weight development of mice treated with 150 mM BA in drinking water ad libitum after Ova/CFA immunization (n = 10). (C) Average daily drinking volume of mice treated with 150 mM BA in drinking water ad libitum after Ova/CFA immunization (n = 10). (TIF) [file pone.0266071.s001.tif]

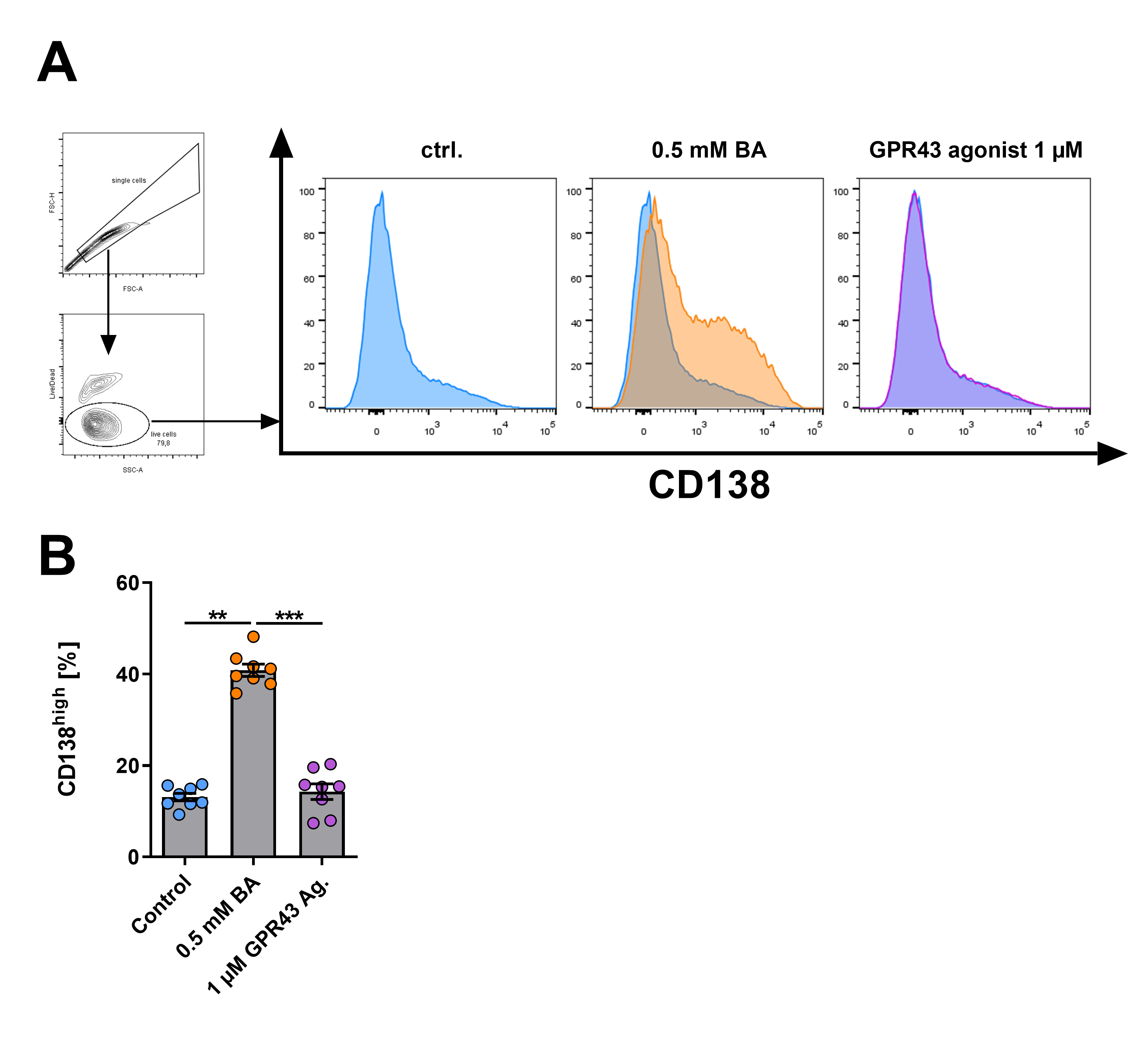

Supplement: S2 Fig — (A) Flow cytometric gating strategy and representative histogram plots for CD138high PCs after treatment with 0.5 mM BA or 1 μM allosteric GPR43 agonist. (B) PC frequencies after treatment of isolated murine B cells with 0.5 mM BA or 1 μM allosteric GPR43 agonist for 4 days of cell culture. ** p < 0.010, *** p < 0.001. (TIF) [file pone.0266071.s002.tif]

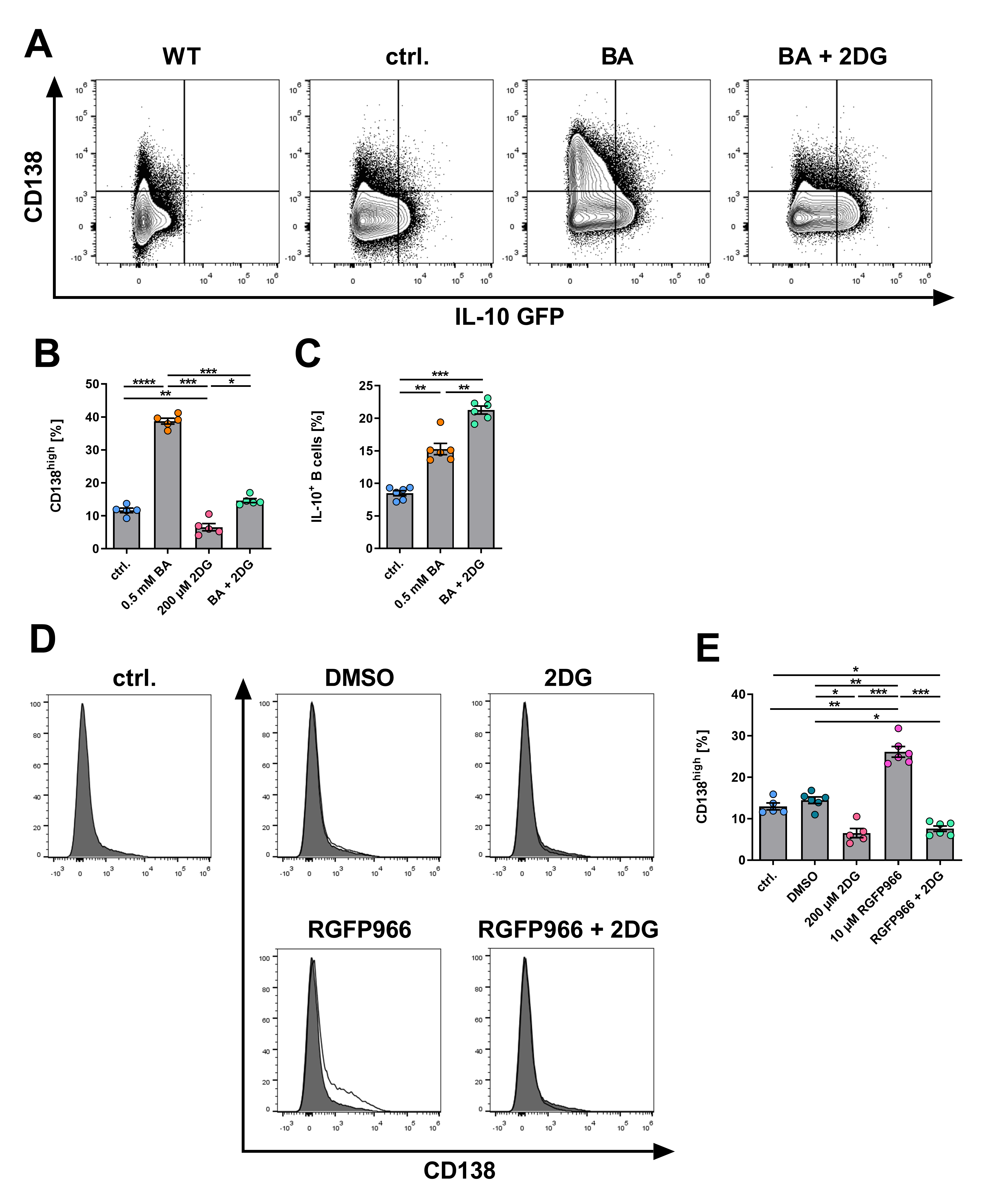

Supplement: S3 Fig — (A) Representative contour plots of CD138 and IL-10 GFP expression in isolated splenic B cells from IL-10 reporter mice after treatment with 0.5 mM BA and 200 μM 2DG. (B) Frequencies of CD138high PCs after treatment of isolated murine B cells with 0.5 mM BA and/or 200 μM 2DG after 4 days of cell culture (n = 5). (C) Frequencies of IL-10+ B cells after treatment of isolated murine B cells with 0.5 mM BA and/or 200 μM 2DG after 4 days of cell culture (n = 6). (D) Representative histogram plots of CD138 expression in isolated splenic B cells after treatment with 10 μM RGFP966 and/or 200 μM 2DG. (E) Frequencies of CD138high B cells after treatment of isolated murine B cells 10 μM RGFP966 and/or 200 μM 2DG after 4 days of cell culture (n = 6). * p < 0.050, ** p < 0.010, *** p < 0.001. (TIF) [file pone.0266071.s003.tif]
